# Non‐communicable disease (NCD) risk among people living with HIV in KwaZulu‐Natal, South Africa: evidence from a randomised trial of community‐based differentiated service delivery

**DOI:** 10.1002/jia2.26513

**Published:** 2025-07-07

**Authors:** Maitreyi Sahu, Adam A. Szpiro, Heidi van Rooyen, Stephen Asiimwe, Maryam Shahmanesh, D. Allen Roberts, Meighan L. Krows, Kombi Sausi, Nsika Sithole, Torin Schaafsma, Jared M. Baeten, Adrienne E. Shapiro, Alastair van Heerden, Ruanne V. Barnabas

**Affiliations:** ^1^ Department of Health Metrics Sciences University of Washington Seattle Washington USA; ^2^ Department of Biostatistics University of Washington Seattle Washington USA; ^3^ Human Sciences Research Council Western Cape South Africa; ^4^ Integrated Community‐Based Initiatives Kabwohe Uganda; ^5^ Africa Health Research Institute KwaZulu‐Natal South Africa; ^6^ Department of Epidemiology University of Washington Seattle Washington USA; ^7^ Department of Global Health University of Washington Seattle Washington USA; ^8^ Division of Allergy and Infectious Diseases University of Washington Seattle Washington USA; ^9^ Gilead Sciences Foster City California USA; ^10^ SAMRC/WITS Developmental Pathways for Health Research Unit Department of Paediatrics School of Clinical Medicine Faculty of Health Sciences University of the Witwatersrand Johannesburg South Africa; ^11^ Human Sciences Research Council KwaZulu‐Natal South Africa; ^12^ Division of Infectious Diseases Massachusetts General Hospital Boston Massachusetts USA; ^13^ Department of Medicine Harvard Medical School Boston Massachusetts USA

**Keywords:** antiretroviral therapy, non‐communicable diseases, cardiovascular risk, community health services, differentiated service delivery, integrated care

## Abstract

**Introduction:**

As differentiated HIV services provided outside of clinics are scaled up, clients may have fewer interactions with ancillary services for non‐communicable disease (NCD) prevention and management traditionally offered within facilities. This study was embedded in the DO ART randomised trial (2016−2019), which demonstrated that community‐based differentiated service delivery (DSD) improved HIV viral suppression compared with facility‐based care. We assessed NCD risk among men and women living with HIV accessing community‐based DSD versus facility‐based care in KwaZulu‐Natal, South Africa.

**Methods:**

First, we described lifestyle and clinical NCD risk among DO ART participants in rural and semi‐rural KwaZulu‐Natal. Next, we compared clinical NCD risk at 12 months by randomisation arm (community‐based DSD vs. facility‐based care). Finally, we explored the relationship between 12‐month viral suppression and clinical NCD risk, overall and stratified by randomisation arm (i.e. service delivery type).

**Results:**

Among 1010 participants, the median age was 32 years, 245 (24%) smoked, 229 (23%) had hypertension and 502 (50%) were overweight or obese (body mass index [BMI] ≥ 25). Smoking was more common among men than women (43% vs. 6%, *p* ≤ 0.001), while overweight/obesity was more common among women than men (65% vs. 34%, *p* ≤ 0.001). We found no statistically significant association between service delivery type and clinical NCD risk factors at 1 year. We also found no significant associations between viral suppression at 12 months and blood pressure, haemoglobin A1c or smoking. However, virally suppressed clients had higher mean BMI (+0.93 kg/m^2^, *p* = 0.004) and higher mean cholesterol (+5.79 mg/dl, *p* = 0.001). These associations were greater in effect size and statistically significant among clients receiving community‐based DSD (BMI: *p* = 0.003; cholesterol: *p* = 0.001), but smaller and not significant for facility‐based care (BMI: *p* = 0.299; cholesterol: *p* = 0.448).

**Conclusions:**

Relatively younger adults accessing HIV treatment in South Africa had substantial NCD risk, which differed by gender and may increase with age. Among clients receiving community‐based DSD, viral suppression was associated with modestly higher BMI and cholesterol levels. Community‐based DSD programmes should consider integrating NCD risk screening and management that addresses gender‐specific needs to prevent premature mortality among people living with HIV.

**Clinical Trial Number:**

NCT0292999

## 1 INTRODUCTION

Sub‐Saharan Africa (SSA) is home to 26 million people living with HIV, nearly 20 million of whom are achieving viral suppression through antiretroviral therapy (ART) [1], but who face a growing burden of non‐communicable disease (NCD) risk [2, 3], especially as the population ages [4, 5]. People living with HIV have an estimated 2.5‐fold higher risk of cardiovascular disease than people without HIV [6]. Moreover, nearly one‐third of people living with HIV in SSA are overweight or obese, one‐fifth have hypertension and one‐fifth have high cholesterol [7].

Differentiated service delivery (DSD)—including both community‐based and facility‐based models—has emerged as a promising strategy for HIV control [8, 9], maintaining or improving ART outcomes [10, 11] while alleviating strain on health systems and patients [12, 13]. However, as these services reduce the frequency of client contact with health facilities [14, 15], clients may have fewer interactions with ancillary clinic services for NCDs. This has important implications, as studies from eastern and southern Africa suggest that individuals receiving ART at clinics may experience *lower* NCD risk than people without HIV—despite the increased cardiometabolic risk associated with HIV acquisition [6, 16] and treatment [17, 18, 19, 20, 21, 22, 23]—due to their engagement with services such as nutritional counselling and NCD management [24, 25, 26].

In response to this dual burden, integrated DSD programmes that address both HIV and NCD risk are being considered [9, 27]. For example, South Africa's Central Chronic Medicine Dispensing and Distribution (CCMDD) programme—serving over four million patients—enables stable individuals to collect medicines for HIV, hypertension and diabetes at shared community pick‐up points [28, 29]. However, in the context of DSD, the burden of NCD risk, how it is affected by community‐based service delivery and its implications are not well understood.

In this study, we report NCD risk among adults accessing community‐based DSD and facility‐based HIV services in South Africa. The first objective was (1) to describe the burden of lifestyle and clinical NCD risk among men and women receiving community‐ and facility‐based ART. Next, we (2) assessed if clients randomised to receive community‐based DSD experienced higher clinical NCD risk after 1 year compared with standard facility‐based care, and (3) evaluated the relationship between effective ART (defined by viral suppression) and clinical NCD risk at 1 year for both models of care. These objectives aim to inform DSD programmes in SSA about NCD risk, gender‐specific strategies for mitigation, and opportunities to integrate NCD prevention and management.

## 2 METHODS

This study was embedded within the DO ART randomised trial in South Africa (2016–2019), conducted in 16 communities in the Northern and Midlands regions of KwaZulu‐Natal [11]. Clients newly initiating HIV treatment were randomised at the household level to one of three arms: (1) clinic‐based ART initiation and follow‐up (standard of care); (2) community‐based ART initiation and follow‐up; or (3) a hybrid model with clinic‐based ART initiation and community follow‐up. The trial results showed that community‐based ART improved viral suppression compared to clinic‐based care, and the hybrid model was non‐inferior to clinic‐based care [11].

Integrated NCD care was not provided in any arm, but NCD risk factors were assessed at baseline and/or exit. Randomisation was independent NCD risk, which did not influence the assignment to study arms. For this analysis, we combined the community and hybrid trial arms — both of which involved follow‐up outside the clinic — and refer to them collectively as “community‐based DSD,” with the standard‐of‐care arm referred to as “facility‐based care.”

### 2.1 Data collection

Data related to NCD risk and HIV clinical outcomes were collected at baseline, study exit (12 months after enrolment) or quarterly visits as follows:

- **Demographics**: Age and gender were recorded at baseline [11].
- **Self‐reported NCD risk factors**: These included smoking status (baseline), weekly exercise (exit), vegetable intake (exit) and history of stroke or heart attack (exit).
- **Clinically measured NCD risk factors**: Trained study nurses collected several clinical NCD measures. For consistency, only exit values were used for analysis.
  - Blood pressure (systolic and diastolic) was measured in triplicate at quarterly visits;
  - Body mass index [BMI] at baseline and exit;
  - Haemoglobin A1c (exit only);
  - Total cholesterol (exit only);
- **HIV clinical outcomes**: Viral suppression, the primary outcome of the DO ART trial, has been previously published [11]. In this analysis, 12‐month viral suppression was examined as a predictor of NCD risk.

### 2.2 Variable definitions

Clinically measured NCD risk factors were categorised using standard guidelines:

- **Blood pressure**. We applied American College of Cardiology/American Heart Association (ACC/AHA) cutoffs using the average of three readings: < 120/80 mmHg (normal), systolic between 120–129 *and* diastolic < 80 (elevated), systolic between 130–139 *or* diastolic between 80–89 (Hypertension Stage 1), systolic ≥ 140 *or* diastolic ≥ 90 (Hypertension Stage 2) and systolic ≥ 180 or diastolic ≥ 120 (Hypertensive crisis) [30].
- **BMI**. BMI ≤ 18.5 kg/m^2^ (underweight), BMI > 18.5 kg/m^2^ and < 25 kg/m^2^, BMI ≥ 25 kg/m^2^ (overweight), BMI ≥ 30 kg/m^2^ (obesity).
- **Glycaemic control**. Haemoglobin A1c < 5.7% (normal), ≥ 5.7% and < 6.5% (prediabetes), ≥ 6.5% (diabetes) [31].
- **Total cholesterol**. < 200 mg/dl (normal), ≥ 200 and < 240 (borderline high), and ≥ 240 (high) [32].

We also dichotomised clinical NCD risk measures into “elevated risk” and “no elevated risk” (with reference categories shown in Tables 2 and 3). Given that only 12 participants had elevated total cholesterol, dichotomised cholesterol level was not included in our analysis.

### 2.3 Analysis

We conducted the following analyses:

1. We described NCD risk (including demographic variables, lifestyle risk factors and clinical risk measures), using chi‐squared tests to assess differences by gender.
2. We compared clinically measured NCD risk factors between participants randomised to receive community‐based DSD versus facility‐based care.
3. We compared clinically measured NCD risk factors between clients who were virally suppressed and those not virally suppressed at 1 year. To assess whether the relationship between viral suppression and NCD risk varied by HIV service delivery type, we conducted:
  - stratified analyses by type of HIV service delivery model, and
  - a pooled analysis including an interaction term between viral suppression status and trial arm (i.e. type of HIV service delivery) to test for statistical evidence of effect modification.

We estimated relative risks [RRs] for binary outcomes using Poisson regression and average (mean) differences [ADs] for continuous outcomes using linear regression. To account for household clustering (1% of individuals in the study [10/1010] were from households with more than one enrolled individual), we used generalised estimating equations with an independence working covariance structure. Unadjusted models included trial site (i.e. Northern or Midlands KwaZulu‐Natal) as the only covariate, while adjusted models also included age (categorical), gender (binary), smoking status (binary) and education level (categorical). Adjusted results are presented as aRRs and aADs throughout the manuscript.

### 2.4 Missing data

Since NCD clinical measures generally had good coverage except for A1c, which was missing from one site due to incorrect test administration, regressions used complete case analysis assuming that NCD data missingness was independent of the outcome, after conditioning on site.

### 2.5 Ethics

All participants provided written informed consent prior to enrolment. The DO ART Study was approved by the Human Sciences Research Council Research Ethics Committee in South Africa and the University of Washington Institutional Review Board, Seattle, WA (REC6/13/11/15).

## 3 RESULTS

Among 1010 eligible participants in the DO ART Study in South Africa, the median age was 32 years (interquartile range: 27–40) (Table 1). Overall, 245 participants (24%) were current smokers (occasional or frequent), 468 (46%) exercised ≤ 2 days/week, 280 (28%) never or rarely ate vegetables and 14 (1%) reported a history of stroke or heart attack. Clinically, 450 (45%) had above‐normal blood pressure including 229 (23%) with Stage 1 or Stage 2 Hypertension, 502 (50%) had BMI ≥ 25, 62 (6%) had prediabetes or diabetes and 12 (1%) had elevated cholesterol.

**Table 1:** Non‐communicable disease risk in the DO ART Study in South Africa, by trial arm and gender (*N* = 1010)

	Category	Community‐based DSD *n* (%)	Facility‐based care *n* (%)	*p*‐value	Women *n* (%)	Men *n* (%)	*p*‐value	Total *N* (%)
**(i) Demographics**								
Age	18−39	490 (74%)	252 (72%)	0.302	386 (76%)	356 (70%)	0.101	742 (73%)
	40−59	164 (25%)	91 (26%)		113 (22%)	142 (28%)		255 (25%)
	60+	6 (1%)	7 (2%)		6 (1%)	7 (1%)		13 (1%)
	Median (IQR)	32 (26–40)	32 (27–40)	–	30 (25–38)	34 (29–41)	–	32 (27–40)
Gender	Women	336 (51%)	169 (48%)	0.467	–	–	–	505 (50%)
	Men	324 (49%)	181 (52%)		–	–		505 (50%)
Education	Primary	121 (18%)	79 (23%)	0.099	74 (15%)	126 (25%)	0.001	200 (20%)
	Secondary	502 (76%)	261 (75%)		406 (80%)	357 (71%)		763 (76%)
	Tertiary and above	21 (3%)	7 (2%)		16 (3%)	12 (2%)		28 (3%)
	Missing	16 (2%)	3 (1%)		9 (2%)	10 (2%)		19 (2%)
**(ii) Self‐reported risk factors**								
Smoking status [Baseline]	Not at all	497 (75%)	261 (75%)	0.429	475 (94%)	283 (56%)	< 0.001	758 (75%)
	Former	3 (0%)	4 (1%)		2 (0%)	5 (1%)		7 (1%)
	Current—occasional	42 (6%)	17 (5%)		13 (3%)	46 (9%)		59 (6%)
	Current—frequent	118 (18%)	68 (19%)		15 (3%)	171 (34%)		186 (18%)
Days of exercise (per week) [Exit]	5−7	38 (6%)	14 (4%)	0.534	13 (3%)	39 (8%)	< 0.001	52 (5%)
	3−4	262 (40%)	136 (39%)		185 (37%)	213 (42%)		398 (39%)
	1−2	187 (28%)	113 (32%)		163 (32%)	137 (27%)		300 (30%)
	0	114 (17%)	54 (15%)		101 (20%)	67 (13%)		168 (17%)
	Missing	59 (9%)	33 (9%)		43 (9%)	49 (10%)		92 (9%)
Vegetable intake [Exit]	Always or usually	122 (18%)	53 (15%)	0.050	103 (20%)	72 (14%)	< 0.001	175 (17%)
	Sometimes	326 (49%)	166 (47%)		266 (53%)	226 (45%)		492 (49%)
	Never or rarely	166 (25%)	114 (33%)		107 (21%)	173 (34%)		280 (28%)
	Missing	46 (7%)	17 (5%)		29 (6%)	34 (7%)		63 (6%)
Prior stroke or heart attack [Exit]	No	606 (92%)	328 (94%)	0.466	469 (93%)	465 (92%)	0.643	934 (92%)
	Yes	9 (1%)	5 (1%)		8 (2%)	6 (1%)		14 (1%)
	Missing	45 (7%)	17 (5%)		28 (6%)	34 (7%)		62 (6%)
**(iii) Clinically measured risk factors**								
Blood pressure [Exit]	Normal	334 (51%)	171 (49%)	0.388	263 (52%)	242 (48%)	0.253	505 (50%)
	Elevated	133 (20%)	88 (25%)		99 (20%)	122 (24%)		221 (22%)
	Hypertension Stage 1	136 (21%)	61 (17%)		104 (21%)	93 (18%)		197 (20%)
	Hypertension Stage 2	20 (3%)	12 (3%)		16 (3%)	16 (3%)		32 (3%)
	Missing	37 (6%)	18 (5%)		23 (5%)	32 (6%)		55 (5%)
BMI (kg/m^2^) [Exit]	< 18.5	20 (3%)	10 (3%)	0.496	4 (1%)	26 (5%)	< 0.001	30 (3%)
	18.5−24.9	268 (41%)	152 (43%)		149 (30%)	271 (54%)		420 (42%)
	25−29.9	219 (33%)	100 (29%)		187 (37%)	132 (26%)		319 (32%)
	30+	113 (17%)	70 (20%)		142 (28%)	41 (8%)		183 (18%)
	Missing	40 (6%)	18 (5%)		23 (5%)	35 (7%)		58 (6%)
Haemoglobin A1C (%) [Exit]	Normal	403 (61%)	235 (67%)	0.236	341 (68%)	297 (59%)	0.013	638 (63%)
	Prediabetes	35 (5%)	19 (5%)		20 (4%)	34 (7%)		54 (5%)
	Diabetes	6 (1%)	2 (1%)		2 (0%)	6 (1%)		8 (1%)
	Missing	216 (33%)	94 (27%)		142 (28%)	168 (33%)		310 (31%)
Total cholesterol (mg/dl) [Exit]	Normal	584 (88%)	324 (93%)	0.109	449 (89%)	459 (91%)	0.550	908 (90%)
	Borderline high	9 (1%)	1 (0%)		7 (1%)	3 (1%)		10 (1%)
	High	2 (0%)	0 (0%)		1 (0%)	1 (0%)		2 (0%)
	Missing	65 (10%)	25 (7%)		48 (10%)	42 (8%)		90 (9%)
**(iv) HIV clinical outcomes**								
Viral suppression^a^ [Exit]	Yes	448 (68%)	204 (58%)	0.007	348 (69%)	304 (60%)	0.014	652 (65%)
	No	194 (29%)	137 (39%)		146 (29%)	185 (37%)		331 (33%)
	Missing	18 (3%)	9 (3%)		11 (2%)	16 (3%)		27 (3%)

*Notes*: *P*‐values are estimated from chi‐squared tests comparing the distribution of each categorical variable across trial arm or gender. Each *p*‐value reflects the overall association across all levels of the variable.

Abbreviations: BMI, body mass index; DSD, differentiatated service delivery; IQR, interquartile range.

^a^ Viral suppression was the primary outcome for the DO ART Study. In South Africa, community‐based DSD was found to significantly increase viral suppression by 1.22 (95% CI: 1.09−1.36) for community‐based initiation and community‐based follow‐up, that is the “community arm” of the trial, and by 1.12 (95% CI: 1.00–1.25) for facility‐based initiation and community‐based follow‐up, that is the “hybrid arm” of the trial [11].

Certain clinical NCD risk factors varied significantly by gender (Table 1). Among men, 217 of 505 (43%) were current smokers, compared to 28 of 505 (6%) of women (*p* < 0.001). For BMI, 173 men (34%) and 329 women (65%) were overweight, including 41 men (8%) and 142 women (28%) classified as obese (*p* < 0.001). Elevated haemoglobin A1c (prediabetes or diabetes) was observed in 40 men (8%) and 22 women (4%) (*p* = 0.013).

Our regression analysis found no significant differences in clinically measured NCD risk factors (blood pressure, BMI, A1c and total cholesterol) between clients randomised to receive community‐based DSD versus those receiving facility‐based care (Table 2).

**Table 2:** Impact of HIV service delivery model on non‐communicable disease risk factors: community‐based differentiated service delivery versus facility‐based care (*N* = 1010)

NCD risk factors		Sample characteristics			Unadjusted analysis			Adjusted analysis		
A. Binary outcomes	Reference category	Total (*n*)	Elevated risk (*n*)	No elevated risk (*n*)	RR	95% CI	*p*‐value	aRR	95% CI	*p*‐value
Above‐normal blood pressure	Normal blood pressure	955	450	505	0.93	(0.79, 1.09)	0.368	0.94	(0.81, 1.10)	0.455
Elevated BMI (BMI ≥ 25 kg/m^2^)	BMI < 25 kg/m^2^	952	502	450	1.03	(0.91, 1.17)	0.633	1.02	(0.89, 1.15)	0.802
Elevated blood sugar (A1c ≥ 5.7%)	A1c < 5.7%	700	62	638	1.22	(0.74, 2.00)	0.440	1.19	(0.73, 1.96)	0.478
Current smoker [baseline]	Non‐/former smoker	1010	245	765	0.99	(0.79, 1.24)	0.919	1.02	(0.81, 1.28)	0.887

B. Continuous outcomes		Total (*n*)	Mean	SD	AD	95% CI	*p*‐value	aAD	95% CI	*p*‐value
Systolic blood pressure (mmHg)		955	116.7	10.2	−0.80	(−2.16, 0.56)	0.247	−0.70	(−2.06, 0.66)	0.306
Diastolic blood pressure (mmHg)		955	74.2	8.3	−0.03	(−1.11, 1.05)	0.956	−0.08	(−1.15, 1.00)	0.888
BMI (kg/m^2^)		952	26.1	5.1	−0.02	(−0.68, 0.64)	0.964	−0.11	(−0.77, 0.55)	0.726
Haemoglobin A1c (%)		700	5.0	0.5	0.04	(−0.04, 0.12)	0.287	0.04	(−0.04, 0.12)	0.278
Total cholesterol (mg/dl)		920	145.6	25.6	3.13	(−0.18, 6.43)	0.064	2.92	(−0.38, 6.22)	0.080

*Notes*: This table shows the randomised comparison of NCD risk for community‐based differentiated service delivery (DSD) compared to facility‐based care. RRs (for dichotomised outcomes) and ADs (for continuous outcomes) were estimated using Poisson and linear generalised estimating equations, respectively. Unadjusted models controlled for trial site only (Northern or Midlands KwaZulu‐Natal). Adjusted models controlled for trial site, age, smoking, gender and education (except the “current smoker” outcome, which did not include smoking). “Above normal” blood pressure includes elevated blood pressure, Stage 1 Hypertension and Stage 2 Hypertension per ACC/AHA guidelines.

Abbreviations: aAD, adjusted average (mean) difference; AD, unadjusted average difference; aRR, adjusted relative risk; BMI, body mass index; CI, confidence interval; RR, unadjusted relative risk; SD, standard deviation.

At 12 months, virally suppressed clients had higher BMI (aAD = +0.94 kg/m^2^, *p* = 0.003), total cholesterol (aAD = +5.71 mg/dl, *p* = 0.001) and risk of overweight/obesity (aRR = 1.16, *p* = 0.024) compared with clients not virally suppressed (Table 3). These associations were not observed for other NCD risk factors, including blood pressure, haemoglobin A1c and baseline smoking. While the interaction term between viral suppression and trial arm (i.e. type of service delivery) was not statistically significant, the patterns differed by service model. In community‐based DSD, virally suppressed persons had significantly higher BMI (aAD = +1.18 kg/m^2^, *p* = 0.003), total cholesterol (aAD = +7.52 mg/dl, *p* = 0.001) and risk of overweight/obesity (aRR = 1.21, *p* = 0.024) compared with clients not suppressed. In contrast, no significant associations were observed among clients in facility‐based care; effect sizes were either attenuated (cholesterol, BMI) or reversed (blood pressure).

**Table 3:** Associations between HIV viral suppression and non‐communicable disease risk factors, stratified by type of HIV service delivery model: community‐based differentiated service delivery (DSD) and facility‐based care (*N* = 983)

NCD risk factors	Community‐based DSD				Facility‐based care				Overall (pooled)				Inter‐action
A. Binary outcomes	*n*	aRR	95% CI	*p*‐value	*n*	aRR	95% CI	*p*‐value	*N*	aRR	95% CI	*p*‐value	*p*‐value
Above‐normal blood pressure	621	1.17	(0.94, 1.46)	0.158	331	0.93	(0.72, 1.19)	0.542	952	1.05	(0.89, 1.24)	0.535	0.148
Elevated BMI (BMI ≥ 25 kg/m^2^)	618	1.21	(1.03, 1.42)	0.024	331	1.08	(0.87, 1.34)	0.489	949	1.16	(1.01, 1.33)	0.024	0.397
Elevated blood sugar (A1c ≥ 5.7%)	443	1.05	(0.57, 1.95)	0.874	255	0.76	(0.32, 1.78)	0.530	698	0.95	(0.58, 1.56)	0.832	0.615
Current smoker [baseline]	642	0.95	(0.72, 1.24)	0.690	341	0.95	(0.67, 1.33)	0.748	983	0.95	(0.75, 1.19)	0.598	0.977

B. Continuous outcomes	*n*	aAD	95% CI	*p*‐value	*n*	aAD	95% CI	*p*‐value	*N*	aAD	95% CI	*p*‐value	*p*‐value
Systolic blood pressure (mmHg)	621	0.94	(−0.70, 2.59)	0.261	331	−1.58	(−3.84, 0.67)	0.169	952	−0.12	(−1.47, 1.23)	0.861	0.051
Diastolic blood pressure (mmHg)	621	0.20	(−1.13, 1.53)	0.767	331	−0.95	(−2.62, 0.73)	0.268	952	−0.25	(−1.33, 0.84)	0.648	0.182
BMI (kg/m^2^)	618	1.18	(0.41, 1.95)	0.003	331	0.56	(−0.49, 1.61)	0.299	949	0.93	(0.28, 1.59)	0.004	0.324
Haemoglobin A1c (%)	443	−0.08	(−0.18, 0.02)	0.118	255	0.06	(−0.07, 0.20)	0.360	698	−0.02	(−0.10, 0.07)	0.709	0.091
Total cholesterol (mg/dl)	593	7.52	(2.93, 12.10)	0.001	324	2.06	(−3.26, 7.39)	0.448	917	5.79	(2.35, 9.23)	0.001	0.115

*Notes*: This table shows the association between HIV viral suppression and NCD risk factors stratified by trial arm, i.e. type of HIV service delivery model. aRRs (for dichotomised outcomes) and aADs (for continuous outcomes) were estimated using Poisson and linear generalised estimating equations, respectively. Adjusted models (all) control for trial site, age, smoking, gender and education (except the “current smoker” outcome, which did not include smoking). The *p*‐value for the interaction term is from a pooled analysis additionally including an interaction term between HIV viral suppression and trial arm. Reference categories for dichotomous outcomes were: normal blood pressure, BMI < 25 kg/m^2^, A1c < 5.7% and non‐ or former smoker. “Above normal” blood pressure includes elevated blood pressure, Stage 1 Hypertension and Stage 2 Hypertension per ACC/AHA guidelines. Twenty‐seven participants without viral load data at exit were excluded from this analysis (*N* = 983).

Abbreviations: aAD, adjusted average (mean) difference; aRR, adjusted relative risk; BMI, body mass index; CI, confidence interval.

## 4 DISCUSSION

In a largely younger cohort (73% aged 18–39) receiving HIV care in rural/semi‐rural South Africa, we observed a substantial burden of clinical NCD risk: 50% had BMI ≥ 25, 45% had above‐normal blood pressure and 24% were current smokers. As this population ages, additional risk factors—such as high cholesterol and diabetes—are also likely to increase [4, 5]. Building on decades of research on gendered patterns of NCD risk [33, 34, 35], we found significant gender differences: 43% of men were current smokers compared with just 6% of women, while 65% of women were overweight/obese compared with 34% of men. Despite the cohort's younger age, the prevalence of smoking, high BMI and blood pressure exceeded average levels in SSA [7], and were comparable to rates observed in the general population in KZN and South Africa [36, 37, 38, 39].

Building on extensive literature linking HIV, ART and effective ART (viral suppression) to increased cardiovascular risk through pharmacologic and metabolic pathways (including pro‐inflammatory effects and treatment‐related weight gain and hyperlipidaemia) [18, 19, 20, 21, 22], we observed several trends relevant to DSD programmes. First, community‐based DSD did not significantly increase NCD risk compared to facility‐based care within 12 months, despite the lack of integrated NCD services. Second, viral suppression was modestly associated with higher BMI and cholesterol in this young population, half of whom were already overweight or obese—an association that may strengthen with expanded use of integrase inhibitor–based ART [21, 40, 41]. Finally, although we did not find statistical evidence of effect modification by the service delivery model, associations between viral suppression and NCD risk were more pronounced in community‐based DSD. This pattern aligns with studies suggesting that access to ancillary services in clinics—including nutritional counselling and preventive medications—may help to mitigate ART‐related NCD risk [24, 25, 26]. These findings highlight the need for community‐based DSD models to prioritise NCD risk screening and management to meet or exceed the standard of care offered in clinics.

Differentiated HIV service delivery models tailor care to the needs of specific populations—including men, women, ageing adults and people with elevated NCD risk. Our findings support this approach by highlighting modifiable risk factors that differ by gender and age. For example, community‐based models designed to engage men in care [11, 42] could incorporate smoking cessation, given that 43% of men in our study smoked and that integrated counselling reduces mortality [43, 44]. Similarly, as DSD increasingly reaches older populations, integrating NCD screening and management will be essential. These adaptations align with emerging guidance on chronic disease integration within DSD [9, 27].

Community‐based platforms offer a strategic opportunity to deliver integrated NCD services. In South Africa, DSD guidance already includes HIV, tuberculosis (TB) and NCD care at both facility and community levels [27, 28]. In our study, conducted before the integration of NCD services into DSD (2016−2019), 21% of clients enrolled in community‐based DSD had Stage I hypertension and 3% had Stage II—but none initiated treatment, highlighting a missed opportunity. South Africa's CCMDD programme, which includes both HIV and NCD medications in its formulary, offers one such integrated model. By requiring in‐person eligibility reassessments every 6–12 months, programmes like CCMDD may provide opportunities to screen for and manage NCD risk over time. In addition, the treatment landscape for NCDs in HIV care is evolving. The recent REPRIEVE trial demonstrated the cardiovascular benefits of statins for people living with HIV [45, 46], while GLP‐1 agonists, widely used for obesity management [47], are also being explored for use among people living with HIV [48]—further strengthening the case for integrated care. However, even inexpensive generic medicines face frequent shortages in low‐ and middle‐income countries [49, 50], and statins and GLP‐1 agonists remain prohibitively expensive for widespread use. Scaling up NCD treatment, including within DSD, will require investment in supply chains to realise the full potential of integrated chronic disease care [51, 52].

Our study had the following limitations. First, our NCD data contained missingness including 31% missing A1c; however, our primary inference relied on measures with less missingness (BMI [3%] and cholesterol [7%]). Second, our analysis did not control for medication use, existing health conditions, diet or alcohol use due to data limitations. Third, most NCD risk factors were measured only at endline, which did not allow for comparisons against the baseline values. Fourth, our exploratory analysis of NCD risk and viral suppression was not randomised and we did not capture long‐term associations; however, the results remained consistent across several robustness checks. Moreover, despite multiple comparisons, the strong statistical significance for BMI and cholesterol suggests robustness to multiple testing. Finally, self‐reported risk is prone to underreporting [53, 54]; however, our main interpretation relied primarily on clinical measures.

## 5 CONCLUSIONS

Our study found substantial NCD risk in a younger population accessing HIV care in South Africa, with clear gender differences and, for community‐based DSD, modest associations between viral suppression and both BMI and cholesterol levels. As HIV services expand beyond clinics, integrating or linking to NCD care will be essential to meeting evolving health needs to prevent premature mortality.

## COMPETING INTERESTS

For work unrelated to the manuscript, AES declares grant funds from Merck as a clinical trials investigator, and RVB reports that Regeneron Pharmaceuticals covered the cost of conference abstract and manuscript writing. RVB serves on a Gilead Sciences DMC for which an honorarium is paid.

## AUTHORS’ CONTRIBUTIONS

MS, AAS and RVB conceptualised the study. MS analysed the data, generated tables and wrote the manuscript. AAS and RVB provided ongoing feedback and critically revised several versions of the manuscript. DAR, AES, TS, JMB and AvH contributed to the study design and interpretation of results. All authors critically reviewed and approved the final manuscript.

## FUNDING

This work was completed with support from the Gates Foundation (BMGF #OPP1134599).

## Data Availability

A de‐identified patient dataset sufficient to reproduce the study findings is available upon request, following approval of a concept sheet with proposed analyses to be done. Further inquiries can be directed to the DO ART Scientific Committee at rbarnabas@mgh.harvard.edu.
